# Reviewed article: Moraes LP, Raposo LM. Impact of Vaccination and SARS-CoV-2 Variants on Severe COVID-19 Outcomes: A Cross-Sectional Study, Brazil, 2021-2022. Epidemiol Serv Saude. 2025:34;e20240613

**DOI:** 10.1590/S2237-96222025v34e20240613.a

**Published:** 2025-09-01

**Authors:** Ernani Tiaraju de Santa Helena

**Affiliations:** 1 Universidade Regional de Blumenau, Blumenau, SC, Brazil Universidade Regional de Blumenau Blumenau SC Brazil

## Peer review A

## Questionnaire

Does the manuscript contain new and significant information to justify publication? No

Does the Abstract (Summary) clearly and accurately describe the content of the article? Yes

Is the problem significant and concisely stated? No

Are the methods described comprehensively? No

Are the interpretations and conclusions justified by the results? Yes

Is adequate reference made to other work in the field? Yes

## Manuscript Structure

Length of article is: Adequate

Number of tables is: Adequate

Number of figures is: Too few

**Please state any conflict(s) of interest that you have in relation to the review of this paper (state “none” if this is not applicable).**

None

**Interest:** Good

**Quality**: Good

**Originality:** Average

**Overall:** Good

**Recommendation:** Major Revision

## Comments

Trata-se de estudo relevante que aborda importante tema para a saúde pública e para a clínica. O texto, no geral, -é bem escrito fundamentado em revisão de literatura atualizada. Os resultados estão apresentados de modo apropriado, articulados aos métodos descritos. A seguir seguem alguns questionamento e sugestões

Resumo

A redação traduz de maneira apropriada os principais resultados e conclusões. No objetivo sugere-se

substituir a expressão “relação” por “associação”. 

Introdução

O contexto está bem colocado. Mas é preciso melhorar a questão que se quer responder, a lacuna de conhecimento. O estado da arte poderia ser mais bem explanado.

Métodos

Participantes: definição, inclusão e exclusão ok

Variáveis: como as variáveis de desfecho são óbito e uso de ventilação mecânica invasiva, por que não foram incluídas potenciais variáveis confundidoras como hospital, cidades/regiões e escolaridade? Incluir referência (ou justificar) as categorias do status vacinal. Os autores consideraram a possibilidade ou adequação de realizar imputação de dados?

Controle de viés: qual a proporção de ausentes? e inconsistentes? As características desses excluídos é semelhante aos inclusos na amostra final? Foram observadas diferenças regionais ou temporais na qualidade dos dados disponíveis? Foram testadas as distribuições das variáveis quantitativas? Uso de Regressão Logística pode ter inflado os resultados da medida de efeito Odds Ratio (em especial quanto aos óbitos) pela elevada frequência dos desfechos. Sugere-se justificar a escolha ou comentar na discussão. Como foi testado o ajuste dos modelos? Descrever. Aspectos éticos: nos parece que a dispensa de aprovação no CEP se dá com base no artigo 1 da resolução CONEP 510/2016

Resultados

Sugere-se evitar a repetição de informações quantitativas que já estão apresentadas nas tabelas (e são muitas repetições!!).

Foram feitas afirmativas de diferenças de proporções entre os grupos. Foram realizados testes que definissem quais grupos diferenciavam-se entre si? Por exemplo, A mediana da duração da hospitalização foi no período da ômicron foi de 7 dias, gama 8 dias e delta 9 dias. Quais são estatisticamente diferentes? Todas? Tabelas: Sugere-se colocar os resultados descritivos e comparativos das variáveis contínuas no corpo do texto ou em figuras (box-plot, p ex.) e deixar nas tabelas essas variáveis como categóricas.

Nas tabelas 1 e 3, para a variável “vacinação contra covid-19” (variável ordinal) o teste de associação foi de tendência? Os autores propõem na tabela 1 uma estratificação da amostra com base nas variantes virais. Na tabela 4

isso se perde! Para melhor entender os desfechos nos períodos sugere-se a separação dos desfechos em 2 tabelas distintas, estratificadas por variante viral.

Discussão

Limitações: não faz menção possível viés de seleção (apesar da amostra de fato ser grande, parece representar algo como 5% do total de SRAG do período estudado). As exclusões e inconsistências que motivaram as exclusões e seu efeito na amostra final não foram mencionadas. Informações sobre regiões/cidades/hospitais/vacinas estão disponíveis no BD original e poderiam ser mencionados as proporções de perdas justificando sua não utilização.

Por vezes a discussão fica confusa. Na pg 8, linha 12 começa a discutir dados não apresentados (interação sexo - idade) que deixa o leitor “às cegas”. Se optar por manter, sugere-se que os resultados sejam apresentados na seção correspondente e não na discussão. Passa a falar de infecções e finaliza o parágrafo falando de superinfecções bacterianas?!

O último parágrafo quase que repete o primeiro parágrafo da discussão. Sugere-se rever a redação.

Referências

Ver normas da revista: as citações devem ser em Vancouver sobrescrito, completo com DOI e link (casodisponível)

Referências atuais (100% menos de 5 anos)

